# Effect of mother’s active pushing at cesarean delivery: a randomized controlled trial

**DOI:** 10.1007/s00404-024-07835-1

**Published:** 2024-11-27

**Authors:** Ahmed Sayed, Anwar A. Sayed, Delnaz Fard, Peter Hillemanns, Constantin Von Kaisenberg, Rüdiger Klapdor

**Affiliations:** 1 Department of Gynecology and Obstetrics, Hannover Medical School, Hannover, Germany; 2 Department of Gynecology and Obstetrics, Taibah University, Madinah, Saudi Arabia; 3 Department of Medical Microbiology and Immunology, Taibah University, Madinah, Saudi Arabia; 4 Department of Surgery and Cancer, Imperial College London, London, UK

**Keywords:** Cesarean delivery, Maternal health, Obstetrics, Family-centered cesarean

## Abstract

**Objective:**

This study aimed to evaluate the effect of maternal active pushing during cesarean section (CS) on postoperative pain, intraoperative discomfort, and the mother’s sense of control and participation.

**Design:**

A prospective, randomized controlled study.

**Methods:**

Patients were randomly assigned into two groups. In the Conventional group (*n* = 45), the CS was performed traditionally without maternal pushing. In the Assisted group (*n* = 55), patients were instructed to push during delivery. Outcomes measures included patients’ perceived pressure, pain, and sense of participation. Breastfeeding and postnatal depression were assessed using validated scales, along with maternal and neonatal outcomes, surgeon satisfaction, and operation duration.

**Results:**

Patients in the Assisted group reported significantly lower fundal pressure intensity (VAS score 3 vs. 5, *P* < 0.01) compared to the Conventional group. There was no significant difference in postoperative pain. However, women in the Assisted group reported a greater sense of participation (6 vs. 0, *P* < 0.01) and control (4 vs. 0, *P* < 0.05) than those in the Conventional group. No significant maternal or neonatal complications were observed.

**Conclusion:**

Maternal active pushing during CS positively impacted intraoperative experience by reducing perceived pressure and enhancing the sense of control and participation, without adverse effects on maternal or neonatal outcomes. These findings support further research with larger, multi-center studies to validate the potential benefits of this approach.

**Trial Registration:**

NCT05520580 (https://clinicaltrials.gov/ct2/show/NCT05520580).

## Introduction

Over the past decades, the rate of cesarean sections (CS) has increased globally. Contributing factors include advancements in antenatal diagnostics, higher rates of multiple pregnancies, and financial and medico-legal considerations [1, 2]. Interestingly, recent data from Germany show a decline in CS rates over the past two years [3]. Current guidelines highlight the disadvantages associated with higher CS rates and generally encourage vaginal delivery when feasible [4]. However, in specific cases, CS remains a necessary intervention to ensure optimal maternal and fetal outcomes [5].

Studies indicate that CS is often associated with lower satisfaction compared to vaginal delivery. A comprehensive review and meta-analysis examining 23 psychosocial outcomes reported that mothers who had CS were less satisfied with the immediate and long-term aspects of delivery, less likely to initiate breastfeeding, experienced delayed interaction with their infants, and had less positive reactions following birth [6]. Another meta-analysis suggested that CS is associated with an increased risk of postpartum depression [7].

A survey involving 1,661 mothers who delivered via CS found that the primary sources of dissatisfaction and distress were poor communication and concerns about missing the birth experience. In contrast, factors like surgical complications and anesthesia had less impact on distress levels [8].

Several interventions, such as “natural” or “gentle” cesarean techniques, have been developed to improve the maternal experience during CS. These approaches allow mothers to observe the birth process and promote immediate or early skin-to-skin contact, aiming to replicate elements of vaginal birth [9, 10]. For instance, Smith et al. introduced the “natural cesarean,” where mothers and partners could view the entire delivery following the hysterotomy, with direct skin-to-skin contact immediately after, with or without active pushing of the mother [11]. Similarly, Armbrust et al. enabled parents to observe the baby’s delivery and participate in cutting the umbilical cord after the baby’s head was delivered [12].

Although such interventions have been shown to enhance maternal satisfaction, they have faced criticism for potentially promoting CS as a viable alternative to vaginal birth, potentially influencing CS rates. Unlike previous studies, our research investigates the feasibility and impact of maternal active pushing during CS. Specifically, we aim to determine if active pushing reduces intraoperative discomfort and postoperative pain and to assess its effect on the delivery process, mother satisfaction, maternal and neonatal outcomes.

## Methods

### Study design

This prospective, randomized study was conducted at Hannover Medical School Hospital from May 2020 to March 2021. The study included patients over 18 years of age undergoing planned CS.

Exclusion criteria were emergency CS, contraindications for epidural anesthesia, multiple pregnancies, planned CS with abnormal placentation, contraindications for the Valsalva maneuver, known psychiatric illnesses, and chronic pain. Non-German-speaking patients were also excluded to maintain consistency in study implementation. A total of 109 patients were recruited, of whom 9 were excluded, resulting in 100 participants randomized into either the Conventional or Assisted groups through simple randomization [13].

### Sample size calculation and power analysis

Given the lack of direct studies on intraoperative pain with maternal active pushing, we referenced related literature. Based on Siddik et al., which reported VAS pain scores with an approximate standard deviation of 2, we assumed a similar standard deviation for our study [14].

We hypothesized that maternal active pushing would result in a clinically significant reduction in intraoperative discomfort, with an expected effect size of 0.8 VAS units between the Assisted (pushing) and Conventional (non-pushing) groups. A two-sided t-test with 80% power at a 0.05 significance level indicated a minimum of 50 participants per group (100 in total) was required. The primary analysis was conducted on an intention-to-treat basis, with conservative imputation methods for minimal missing data, ensuring robust evaluation of the intervention’s effects.

### Intervention

Three surgeons trained in the procedure performed the CS. They were informed about the randomization outcome just prior to surgery and were instructed to maintain similar levels of intraoperative communication in both groups. Anesthetists followed a standardized protocol for intraoperative pain management.

The primary difference between groups occurred during delivery. In the Conventional group, the mother was informed when the baby was about to be delivered, and fundal pressure was applied without maternal pushing. In the Assisted group, after hysterotomy, mothers were instructed and encouraged to push during delivery of the baby’s head and shoulders, with surgical assistants applying fundal pressure as needed. Early skin-to-skin contact was provided in both groups following our center’s routine protocol (Table 1).

**Table 1:** Summary of the study protocol

Procedures	Conventional group	Assisted group
Preoperative preparation	Routine epidural anaesthesia is performed, and sterile preparation is carried out The partner is allowed to enter the operating room and join his partner	
Predelivery surgical procedure	The Misgav–Ladach technique is used to open the abdomen Sharp entry through Pfannenstiel incision	
	A horizontal sharp incision on the lower uterine segment, the surgeon lifts the head of the baby out of the uterus	
Delivery of the baby	Delivery of the baby is done routinely with the assistant giving fundal pressure	Ask the mother to push for completing the delivery of the baby. Gentle help from the surgeon may be needed after delivering the shoulder of the baby
Postdelivery surgical procedure	The surgeon will show the baby to partners and after that, the midwife will take the baby to the examination room then bring back the baby to her mother to allow early skin-to-skin contact	
	The operation is completed in the same way in both groups	
Postoperative interview	Both groups will be interviewed and be asked to fill out the questionnaire at 0,1 and 2. Postoperative day	

### Measurements

Primary outcomes included intraoperative pressure and postoperative pain, assessed on a 10-point Likert scale at four time points: immediately post-delivery (intraoperative), 4–6 h post-delivery, and on the first and second postoperative days. Pain management protocols were identical across groups.

Secondary outcomes included patient satisfaction, breastfeeding, operation duration, surgeon experience, and maternal and neonatal outcomes. A Likert scale-based questionnaire, adapted from previous studies [10, 12, 15], evaluated maternal satisfaction and surgeon experience. We also used the Breastfeeding Self-Efficacy Scale-Short Form (BSES-SF) [16] and the Edinburgh Postnatal Depression Scale (EPDS) [17] for evaluating breastfeeding confidence and psychological status. Neonatal outcomes included Apgar scores, umbilical cord analysis, and NICU admission. Maternal outcomes encompassed estimated blood loss, intraoperative injuries, ICU admission, and hospital stay duration.

### Statistical analysis

Data analysis was performed using Microsoft Excel 2010 (Microsoft Corp., Redmond, WA, USA), IBM SPSS version 24 (IBM Corp., Armonk, NY, USA), and GraphPad Prism 9.3 (GraphPad Software, San Diego, CA, USA). The Shapiro–Wilk test assessed data distribution. Parametric data were analyzed with an independent two-tailed t test, nonparametric data with the Mann–Whitney *U* test, and categorical variables with the Chi-square test. Spearman correlation was used to assess relationships between variables. A *P* value of < 0.05 was considered statistically significant.

## Results

### Patient characteristics

**Fig. 1:**
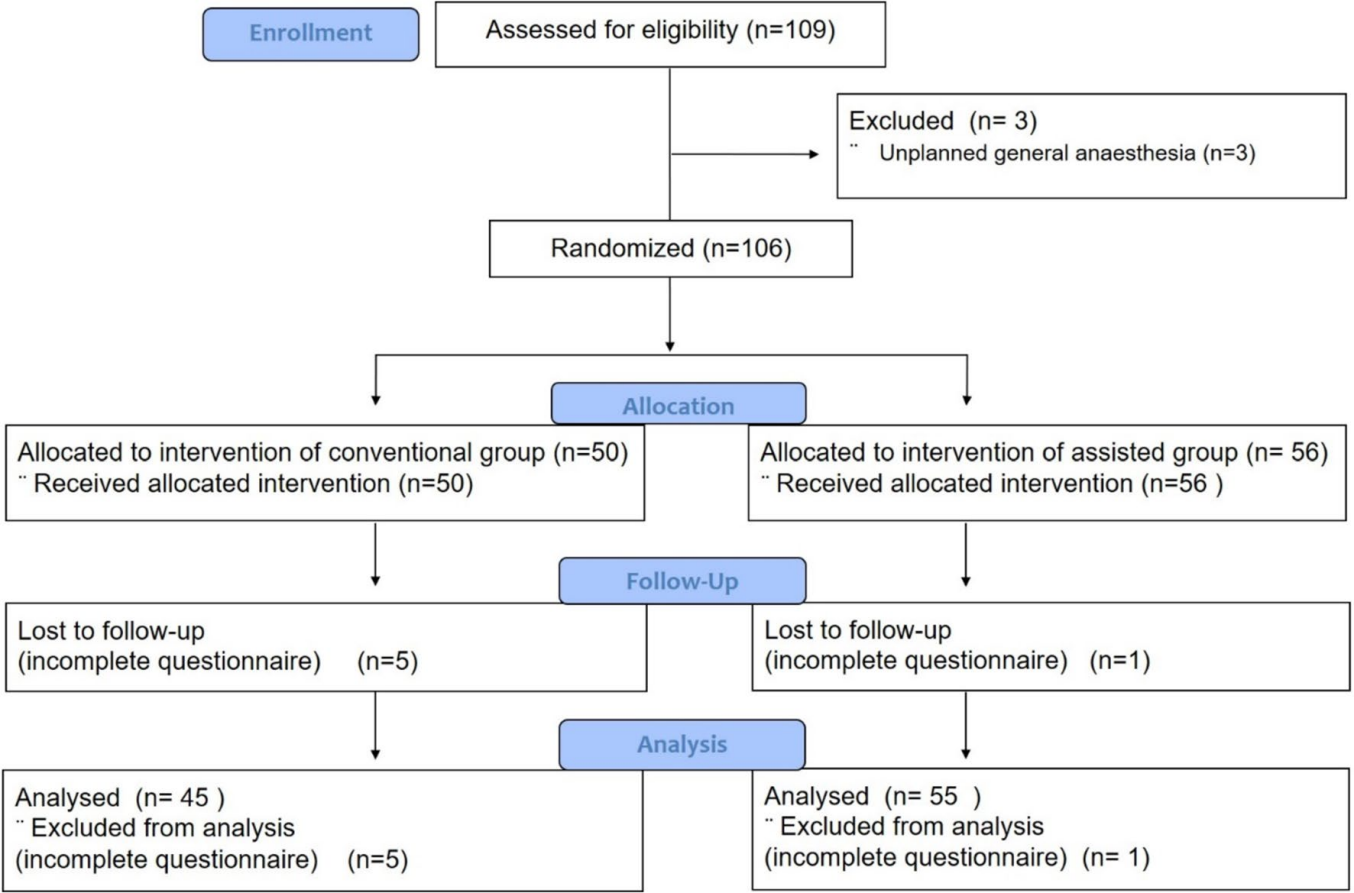
Flowchart of the patient’s recruitment. A PRISMA flow diagram demonstrating the process of recruiting the study subjects

109 patients were recruited, six patients did not return all questionnaires and three had to undergo unplanned general anaesthesia (Fig. 1). The 100 patients were randomized into two groups, the assisted group (*n* = 55) and the conventional group (*n* = 45). The median age was 34 (range 24–44) years and 33 (range 24–43) years in the assisted and conventional groups, respectively. The median parity was 2 (0–4) and 1 (0–3) in the assisted and conventional groups, respectively. There were no significant differences regarding patients’ characteristics as summarized in Table 2. Indications for CS among participants included previous CS, fetal macrosomia, maternal request, malpresentation, large fibroids, maternal disease, and small for gestational age.

**Table 2:** Patient’s characteristics

Patients’ characteristics	Assisted	Conventional	P value
Age: median (range) years	34 (24–44)	33 (24–43)	0.20
BMI	32	29	0.13
Gravida: median (range)	2 (1–4)	2 (1–4)	0.78
Para: median (range)	2 (0–4)	1 (0–3)	0.06
Indication of CS: Malpresentations	10	7	0.76
Fetal macrosomia	2	0	
previous CS	39	31	
Others*	4	6	

Age was compared using the *T* test, while Para and gravida were compared using the Mann–Whitney *U* test. Characteristics were compared using the Chi-square test. *Others include: Fibroid, CS on maternal request, mother disease and small for gestational age

### Assisted CS delivery resulted in lower intraoperative pressure

**Fig. 2:**
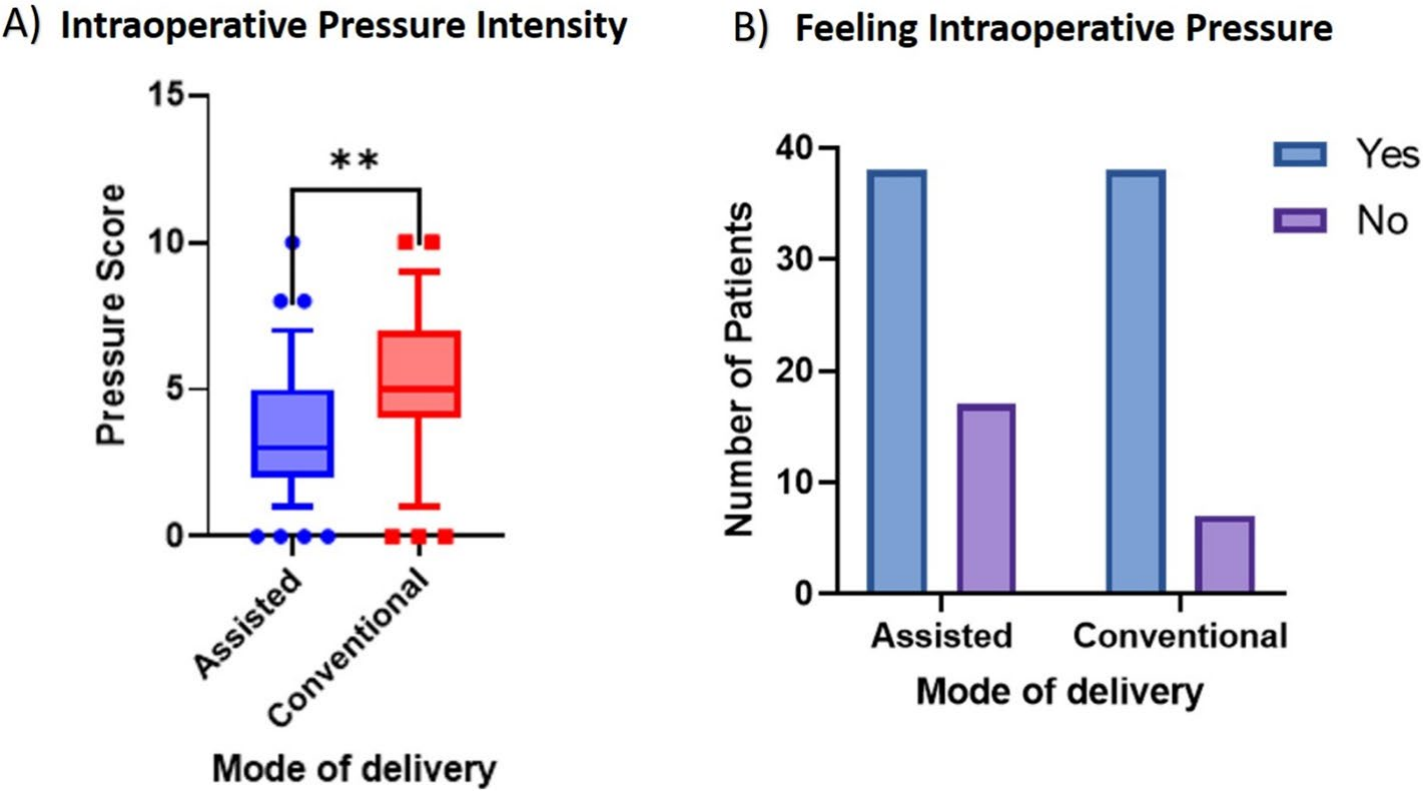
Comparison of intraoperative pressure feeling. Subjects were compared based on (**A**) whether they sensed any pressure intraoperatively (Blue columns) or not (Purple columns) between the groups. Then, subjects (**B**) rated the intensity of the intraoperative pressure sensed, in both the assisted (blue box) and conventional group (red box). Figure B demonstrates the values in a box (lined by the median) and interquartile range. **P value < 0.01

**Fig. 3:**
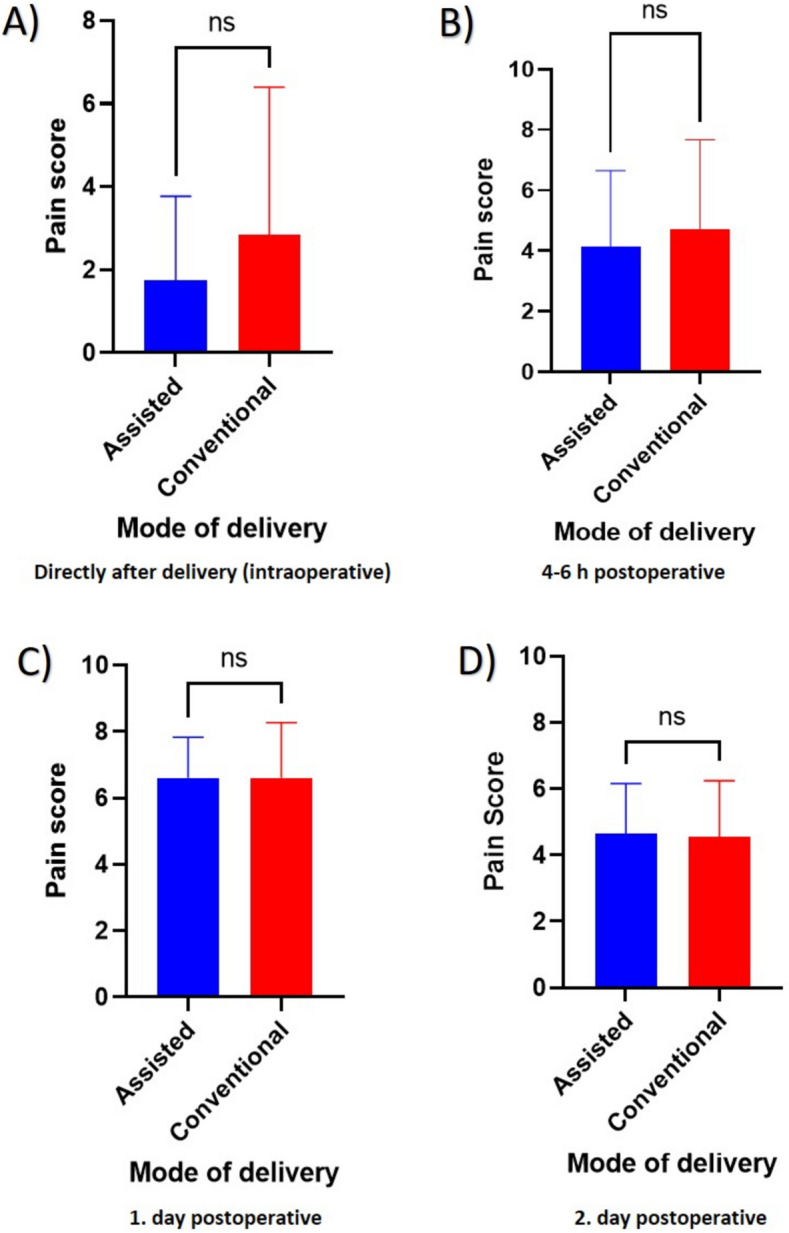
Comparison of the pain measured at different time points. The figure demonstrates the pain intensity measured (**A**) intraoperatively, (**B**) 4–6 h postoperatively, (**C**) 1-day postoperatively and (**D**) 2 days postoperatively. The comparison shows non-significant differences between the assisted (blue) and conventional (red) groups. ns: non-significant

Women actively pushing during delivery reported significantly reduced intensity of intraoperative discomfort (3 vs 5; *P* value < 0.01) compared to the conventional group (Fig. 2A). About 30.9% of the mothers in the assisted group, compared to 15.5% in the conventional group, did not feel pressure during the operation at all (Fig. 2B). We detected no significant differences related to the primary endpoint at different time points in the two groups (Fig. 3).

### Assisted CS delivery improved the senses of participation and control during the surgery

**Fig. 4:**
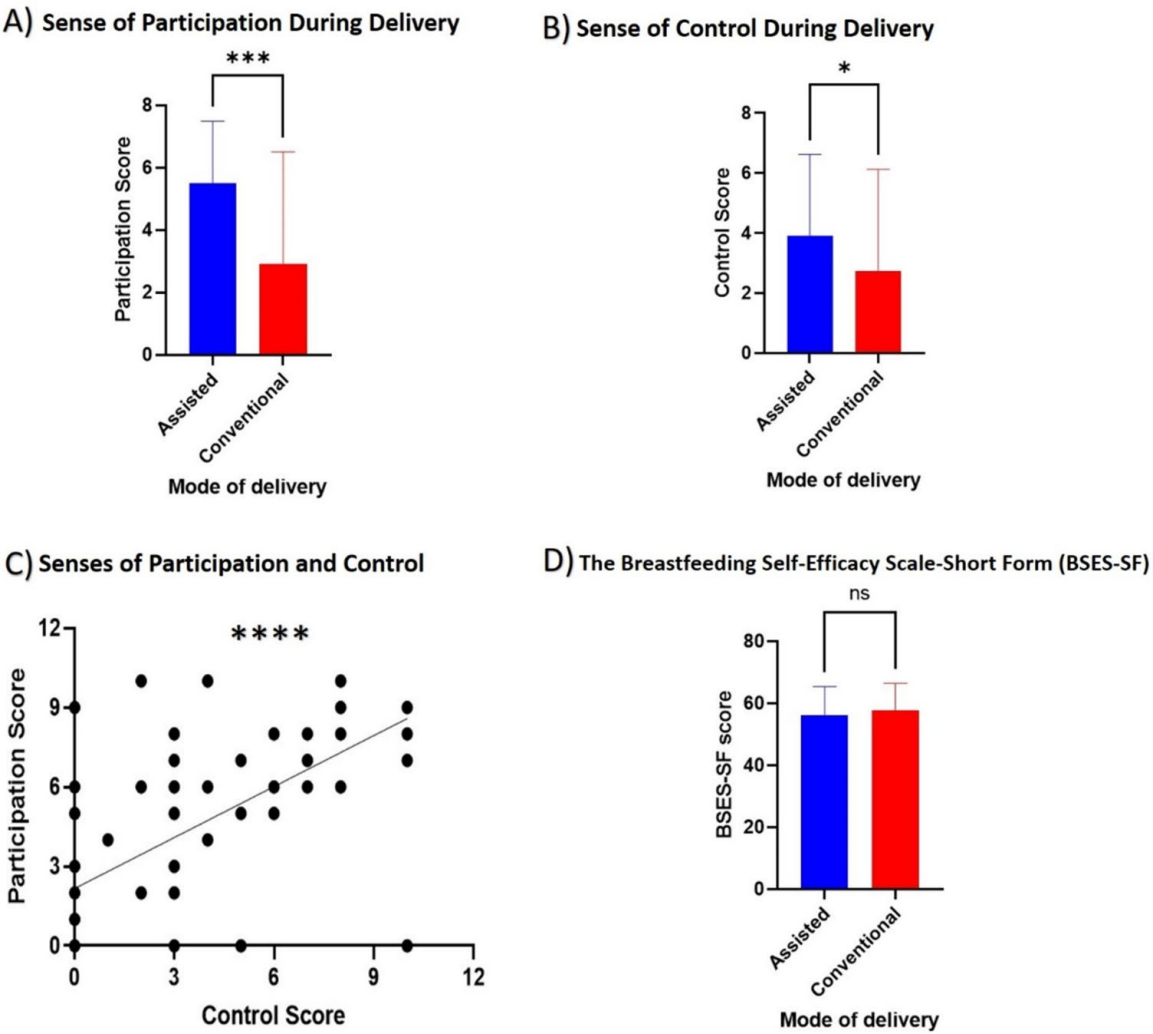
Comparison of the senses of participation and control during the operation and the breastfeeding efficacy. The figure demonstrates comparisons of (**A**) sense of participation and (**B**) control during the delivery, showing the assisted group (blue) had a significantly higher sense of control and participation as compared to the conventional (red) group. (**C**) The significant positive correlation between the senses of participation and control among the assisted group. (**D**) the breastfeeding efficacy as measured by the BSES-SF shows no significant difference between the assisted and conventional groups. *P value < 0.05, ***P value < 0.001, ****P value < 0.0001, ns: non-significant

Allowing mothers to participate during the surgery had a positive effect on the feeling of participation and the sense of control. Mothers in the assisted group reported a significantly higher feeling of participation (6 vs 0, *P* value < 0.01) and sense of control (4 vs 0, *P* value < 0.05) when compared to those in the conventional group (Fig. 4A–B). Interestingly, there was a significant positive correlation between the sense of participation and control among the assisted group (Fig. 4C).

However, no differences were observed between the two groups related to BSES-SF as the early skin-to-skin contact was done similarly in the two groups (Fig. 4D). Similarly, no noticeable differences were observed between the two groups related to EPDS (*P* value 0.84) (Table 3).

**Table 3:** Postnatal depression in the study subjects

C/S–EPDS	Not likely depression (< 9.5)	Intermediate possibility (9.5–10)	Possible depression (> 10)
Assisted	42	2	1
Conventional	43	1	1

The Chi-square test was used to compare the two groups (*P* value 0.84)

*C/S* Caesarean section, *EPDS* Edinburgh Postnatal Depression Scale

No significant maternal or neonatal complications were reported (Table 4), No neonatal respiratory assistance nor neonatal intensive care unit Admission were recorded in both groups (Table 5).

**Table 4:** Maternal and neonatal outcome

Measurement	Assisted	Conventional	*P* value
Estimated blood loss (ml) Median (+ IQR)	400 (300–500)	400 (300–500)	0.76
Surgical injuries	0	0	> 0.99
ICU Admission	0	0	> 0.99
Duration of hospital stay (days)	3 (2–4)	3 (2–5)	0.14
NICU Admissions	0	0	> 0.99
Apgar score 1 Median (+ range)	9 (7–9)	9 (8–9)	0.01*
Apgar score 5 Median (+ range)	10 (8–10)	10 (9–10)	> 0.99
Apgar score 10 Median (+ range)	10 (9–10)	10 (10–10)	> 0.99
Umbilical artery pH Median (+ IQR)	7.3 ( 7.28- 7.31)	7.3 (7.29–7.32)	0.12
Umbilical vein pH Median (+ IQR)	7.33 ( 7.32- 7.35)	7.33 ( 7.31- 7.35)	0.47
Umbilical artery BE Median (+ IQR)	− 2.00 (− 2.8–− 1.3)	− 2.00 (− 3.2–− 1.2)	0.77
Umbilical vein BE Median (+ IQR)	− 1.2 (—2.2–− 0.2)	− 1.3 (− 3.1–− 1.0)	0.2

The table demonstrates the different measurements regarding the maternal and the neonatal outcome. These measurements include estimated blood loss, Surgical injuries, Intensive care unit (ICU) Admission, duration of hospital stay, neonatal intensive care unit (NICU) Admissions, Apgar scores at minutes 1, 5 and 10, umbilical artery and vein’s pH and BE. Mann–Whitney *U* test was used to compare between these parameters

*Denotes statistical significance at *P* value < 0.05

*IQR* Interquartile Range, *BE* Base Excess, *pH* potential of Hydrogen, *ICU* Intensive Care Unit, *NICU* Neonatal Intensive Care Unit

**Table 5:** Results of the surgeons’ experience

Surgeon’s parameter	Assisted	Conventional	P value
Satisfaction (Median + IQR)	8 (7–10)	8 (7.50–9.50)	0.98
Communication with the patient (Median + IQR)	9 (8–10)	9 (7–10)	0.79
Ease of Baby’s Extraction (Median + IQR)	8 (5–9)	8 (5–10)	0.46
Intensity of Fundal Pressure (Median + IQR)	5 (3–7)	5 (4–6)	0.33
Total Duration of Operation [Median minutes (IQR)]	41 (35–47)	40 (36–48)	0.78
Time Between Incision and Delivery [Median minutes (IQR)]	7 (5–8)	7 (5–8.75)	0.73

Mann–Whitney *U* test was used to compare between these parameters

*IQR* Interquartile Range

### Assisted CS delivery did not influence the duration of the procedure

A form was filled out after every operation to assess the mother participation from the surgeons’ point of view. There were no significant differences between the two groups concerning the general satisfaction with the operation, the ease of delivery and the level of communication with the patient Table 5.

We calculated the duration of the operation and the time required from the beginning of the operation until the delivery of the baby. There was no significant difference between the two groups (41 vs 40 min, *P* value = 0.78).

## Discussion

This study offers valuable insights into the effects of active maternal pushing during cesarean section (CS) on maternal comfort, psychological outcomes, and the overall delivery process. These findings are particularly relevant in the ongoing debate about the optimal mode of delivery and the implications of rising CS rates worldwide [4].

One of this study’s key findings is that active maternal pushing during CS significantly reduces intraoperative discomfort. Women who were encouraged to push during delivery reported lower discomfort levels compared to those in the conventional CS group. This supports the hypothesis that active participation during delivery can enhance maternal comfort by providing a sense of control and involvement, an idea previously suggested in studies on vaginal delivery [4].

This psychological benefit is crucial, as prior research has shown that feelings of control and involvement are vital for positive birth experiences [4, 6, 18]. While it may seem intuitive that maternal involvement during cesarean delivery would enhance psychological outcomes, empirical data to confirm this hypothesis have been sparse [8, 18]. This study aims to fill this gap by providing quantifiable evidence of the impact of maternal pushing on intraoperative experiences and to support its application in clinical routine.

However, it is important to note that despite these psychological benefits, the intervention did not significantly influence other outcomes, such as postoperative pain, breastfeeding efficacy (measured by the BSES-SF), or the risk of postpartum depression (EPDS scores). Furthermore, while maternal pushing during CS was associated with improved feelings of control and participation, it is possible that these benefits may partially reflect a placebo effect. The mere act of engaging mothers in the process may create a perception of involvement and control, without necessarily leading to physiological improvements. This raises the question of whether this intervention should be adopted as a routine practice.

Nevertheless, even if a placebo effect plays a role, it still has clinical relevance. In many cases, the perception of control and participation can lead to improved maternal satisfaction, regardless of whether there are direct physiological changes. Given the psychological importance of birth experiences, particularly in the context of cesarean sections, the subjective benefits of maternal pushing may still warrant consideration as part of the birth process, especially in settings where enhancing maternal well-being is a key priority.

Importantly, the current study addresses a critical concern in the field: the need to improve the maternal birth experience during CS without inadvertently promoting CS as an alternative to vaginal birth. By focusing on a specific intervention within the CS process, rather than advocating for CS in general, this study effectively navigates this concern. The findings could inform guidelines and protocols aimed at optimizing CS and improving maternal outcomes without contributing to an increase in overall CS rates.

This study introduces a practical approach that can be easily implemented in various hospital settings, aiming to minimize potential negative effects of routine CS. Encouraging active maternal pushing during CS may reduce feelings of fear by enhancing their sense of participation and control. This study did not show any negative effects on maternal or fetal outcomes, nor did it prolong surgery times, underscoring the safety and feasibility of this approach.

CS are a cornerstone of obstetric practice, despite their reported side effects [4]. Continuous efforts have been made to improve the patient experience during CS, such as allowing patients to watch the delivery after the uterotomy or enabling early skin-to-skin contact [10]. Other interventions include dimming operating theatre lights and playing music during the procedure [12]. These approaches, although varied, share the common goal of enhancing the maternal birth experience during CS [9–12].

Notable attempts to improve CS experiences include the “Natural Caesarean,” introduced in 2008, which allowed parents to observe the delivery of the baby without surgical intervention [11], and the “Charité Caesarean Birth,” published in 2016, which enabled spontaneous delivery of the baby after the head was delivered [12]. Both studies reported improvements in patient birth experiences [11, 12], though the specific factors contributing to this improvement were not clearly identified, as these procedures incorporated multiple elements such as partner participation and environmental modifications.

A recent randomized controlled trial attempted to enhance the CS experience by allowing patients to watch the delivery through a transparent layer, a method termed “family-centred” cesarean. While the study found that the time to skin-to-skin contact was significantly lower in the family-centred group, there were no significant differences in maternal satisfaction, blood loss, or neonatal outcomes [10].

It has to be critically stated that, in these studies, more variables were added to the experimental group, whether before or during the operation, which are likely to have affected the scientific outcome. Additionally, enabling mothers to see inside their abdomens was essential in some of the previous studies, and this matter may have different effects on patients and may give counterproductive results in the long run, especially when the CS is considered a stressful event to many mothers [19].

While it may seem intuitive that maternal pushing during cesarean enhances feelings of participation and control, this study is among the first to quantitatively assess this effect in a structured, randomized manner. Previous literature on maternal involvement in cesarean delivery has focused on interventions like watching the baby’s birth or facilitating early skin-to-skin contact [11, 12]. However, the active physical participation of the mother during the surgical delivery has not been rigorously evaluated, making these findings novel in this context.

Such findings are crucial as they offer practical, evidence-based strategies for improving maternal satisfaction during cesarean deliveries.

As the World Health Organization (WHO) recommends skin-to-skin contact at birth regardless of delivery method, except when separation is medically necessary, early skin-to-skin contact should be a routine part of every cesarean section, regardless of any new changes or approaches to CS [20].

## Limitations

One limitation of this study is the absence of multivariable analysis to control for potential confounders. Although randomization was employed to minimize bias between groups, certain variables (e.g., maternal age, parity, and other clinical characteristics) may still have influenced the outcomes.

Additionally, the assessment of maternal psychological state using the Edinburgh Postnatal Depression Scale (EPDS) and breastfeeding efficacy with the Breastfeeding Self-Efficacy Scale-Short Form (BSES-SF) was conducted over a short-term period, which may not fully capture these dimensions. Including patients with CS categories 2 and 3, as defined by the Royal College of Obstetricians and Gynaecologists (RCOG) classification, would also enrich future research and broaden the applicability of the results [21]. Finally, as this study was conducted at a single center, its generalizability may be limited. Future multi-center studies with larger sample sizes are recommended to confirm and extend our findings.

## Conclusion

This study is the first prospective, randomized trial to examine the effects of maternal active pushing during CS. While no significant differences were observed in postoperative pain, maternal active participation was associated with positive effects on intraoperative experience, including reduced perceived pressure and increased feelings of control and participation. Importantly, there were no adverse effects on maternal or neonatal outcomes, nor was there an impact on the duration of the operation. These findings suggest that maternal involvement during CS improve the birth experience and should be further explored in larger, multi-center trials to assess its broader potential benefits.

## Data Availability

The datasets used and/or analyzed during the current study are available from the corresponding author on reasonable request.

## Abbreviations

CS: Cesarean section
VAS: Visual Analog Scale
BSES-SF: Breastfeeding Self-Efficacy Scale-Short Form
EPDS: Edinburgh Postnatal Depression Scale
PH: Potential of hydrogen
BE: Base excess
WHO: World Health Organization
RCOG: The Royal College of Obstetricians and Gynaecologists
